# Genetic Polymorphisms at TIMP3 Are Associated with Survival of Adenocarcinoma of the Gastroesophageal Junction

**DOI:** 10.1371/journal.pone.0059157

**Published:** 2013-03-19

**Authors:** Morteza Bashash, Amil Shah, Greg Hislop, Martin Treml, Karla Bretherick, Rozmin Janoo-Gilani, Stephen Leach, Nhu Le, Chris Bajdik, Angela Brooks-Wilson

**Affiliations:** 1 Cancer Control Research Program, British Columbia Cancer Agency, Vancouver, British Columbia, Canada; 2 Interdisciplinary Oncology Program, University of British Columbia, Vancouver, British Columbia, Canada; 3 Medical Oncology, British Columbia Cancer Agency, Vancouver, British Columbia, Canada; 4 Department of Medicine, University of British Columbia, Vancouver, British Columbia, Canada; 5 Canada’s Michael Smith Genome Sciences Centre, British Columbia Cancer Agency, Vancouver, British Columbia, Canada; 6 School of Population and Public Health, University of British Columbia, Vancouver, British Columbia, Canada; 7 Department of Statistics, University of British Columbia, Vancouver, British Columbia, Canada; 8 Department of Biomedical Physiology and Kinesiology, Simon Fraser University, Burnaby, British Columbia, Canada; Sanjay Gandhi Medical Institute, India

## Abstract

The poor survival of adenocarcinomas of the gastroesophageal junction (GEJ) makes them clinically important. Discovery of host genetic factors that affect outcome may guide more individualized treatment. This study tests whether constitutional genetic variants in matrix metalloproteinases (MMP) and tissue inhibitors of metalloproteinases (TIMP) genes are associated with outcome of GEJ adenocarcinoma. Single nucleotide polymorphisms (SNPs) at four TIMP (*TIMP1*-*4*) and three MMP genes (*MMP2*, *MMP7* and *MMP9*) were genotyped in DNA samples from a prospective cohort of patients with primary adenocarcinoma of the GEJ admitted to the British Columbia Cancer Agency. Cox proportional hazards regression, with adjustment for patient, disease and treatment variables, was used to estimate the association of SNPs with survival. Genotypes for 85 samples and 48 SNPs were analyzed. Four SNPs across *TIMP3*, (rs130274, rs715572, rs1962223 and rs5754312) were associated with survival. Interaction analyses revealed that the survival associations with rs715572 and rs5754312 are specific and significant for 5FU+cisplatin treated patients. Sanger sequencing of the *TIMP3* coding and promoter regions revealed an additional SNP, rs9862, also associated with survival. *TIMP3* genetic variants are associated with survival and may be potentially useful in optimizing treatment strategies for individual patients.

## Introduction

Adenocarcinomas of the lower esophagus and the proximal stomach (known as gastroesophageal junction or GEJ adenocarcinomas) [1] are rare, deadly and relatively under-studied. During the past two decades, there has been a dramatic increase in the incidence of adenocarcinoma of both the esophagus and proximal stomach in North America and Western Europe [2]–[5].

Tumor cells can interact with surrounding cells to create an environment that can promote tumor growth and protect the tumor from immune attack [6]. The extracellular matrix (ECM) influences tissue and organ architecture, as well as the growth of neoplastic cells [7]. Matrix metalloproteinases (MMPs) are ECM proteases that have been implicated in carcinogenesis and metastasis [8]. MMPs can be synthesized by tumor cells, but are frequently produced by surrounding stromal cells, including fibroblasts and infiltrating inflammatory cells [9]. They can influence cellular properties such as growth, death and migration and contribute to the invasion, promotion, angiogenesis, and the establishment and growth of metastatic lesions in distant organ sites [9]. The balance between activated matrix metalloproteinase (MMP) and tissue inhibitors of metalloproteinase (TIMP) controls ECM remodelling [10], making both TIMPs and MMPs rational candidate genes for cancer outcome studies.

The objective of this study was to assess genetic polymorphisms at specific TIMP and MMP genes for association with outcome for patients with adenocarcinomas of the esophagus and GEJ. All four members of TIMP gene family (*TIMP1* to *TIMP4*) were chosen as candidate genes, due to their roles as key regulators of ECM remodelling. MMPs comprise a large gene family of at least 25 members. To limit the number of tests and preserve statistical power, we chose three MMP genes (*MMP2*, *MMP9* and *MMP7*), for which polymorphisms in the promoter region have been previously associated with gastric and esophageal cancers [11].

## Materials and Methods

The study was approved by the University of British Columbia/British Columbia Cancer Agency (BCCA) Joint Research Ethics Board. All subjects provided written informed consent.

This study used a prospective cohort of patients diagnosed with primary adenocarcinoma of the GEJ between January 1, 2008 and April 30, 2009; admitted to the BCCA in British Columbia (BC), Canada; and able to provide written informed consent. Patients were identified using electronic appointment list and pathology reports of the BCCA Gastrointestinal (GI) Tumor Group for new gastric and esophageal cancer patients. The anatomic sites esophagus and cardia were defined as International Classification of Diseases for Oncology (ICDO-3) site codes C150–C160; adenocarcinoma was defined as ICDO-3 histology codes 8140/3–8573/3. Eligibility and capability to participate were assessed by a BCCA GI Tumor Group oncologist.

DNA was obtained from whole blood (47 patients) or saliva collected using Oragene® DNA sample collection kits (47 patients). To eliminate bias due to the ethnically heterogeneous BC population only the 90 patients who identified themselves as white Canadian, British, or Western European were included in genotyping.

### Clinical Data Collection

Patient characteristics and clinical information were obtained from BCCA medical charts and pre-admission questionnaires. Patient age was categorized as <65 or 65+ based on the median age of patients. BMI was categorized into three groups (normal: 18.5 to <25, overweight: 25 to <30, and obese: 30+). Disease stage was defined according to recent American Joint Committee on Cancer Guidelines [12] and categorized as metastatic or non-metastatic cancer. The Gastrointestinal Tumour Group at the BCCA provides care for all patients in the province, including all participants in this study, and uses province-wide treatment guidelines and protocols (http://www.bccancer.bc.ca/HPI/CancerManagementGuidelines/Gastrointestinal/default.htm). Treatment was categorized as chemotherapy (5-fluorouracil[5FU] and cisplatin), radiotherapy (4500 centi-Gray in 25 fractions), or surgery, with only therapeutic surgeries considered as treatment. Overall survival was the primary study outcome, and was calculated as the time between diagnosis and death. April 30, 2010 was the end of follow-up; by this date all patients had at least 1 year of follow-up information. Median follow-up was 16.7 months.

### SNP Selection

TagSNPs [13] representing genetic variation in each gene were chosen using Haploview version 4.1 [14] on HapMap [15] (phase 3 release 2) western European ancestry (CEU) data. TagSNPs with a minimum minor allele frequency (MAF) of 0.1 were chosen within 10 kb of each gene using an r^2^ threshold of 0.9. Non-synonymous coding SNPs, and SNPs reported in the literature to be associated with cancer, were force-included in the tagSNP selection. The MAF values of these SNPs were obtained using HapMart (BioMart version 7 using HapMap release 27) on CEU population data. The list of SNPs genotyped is in **Table S1**.

### Genotyping and Quality Control

Ninety subject DNA samples were genotyped for 63 SNPs using two multiplex Sequenom iPLEX Gold assays [16] at the McGill University/Genome Quebec Innovation Centre. 88 out of 90 DNA samples (98%) produced genotypes; 3 samples with call rates <95% were excluded, leaving 85 samples for analysis. For quality control of SNPs, the clustering of observed genotypes was reviewed manually by transferring intensity data to MassArray Typer software (version 7.0.2.5). Fifteen SNPs with call rate <95% were excluded from analysis. The average call rate of the remaining 48 SNPs was 98%. Concordance between 2 pairs of duplicate samples was 100% for all SNPs. The genotypes of X chromosome SNPs (rs6609533, rs4898) were consistent with the recorded sex of the patients. 85 samples and 48 SNPs across 7 genes were used for analysis.

### Sequencing Exons of *TIMP3*

To identify possible functional genetic variation, the coding region, 5′ untranslated region and the promoter region of the *TIMP3* gene were sequenced in all 90 European-ancestry GEJ adenocarcinoma patients. Approximately 7700 bp in 15 amplicons were sequenced using Sanger sequencing methods described previously [17]. Primer sequences and PCR conditions are in **Table S2**.

### Statistical Analysis

Survival estimates were calculated using the Kaplan-Meier method; log-rank tests were used to compare survival differences. Haplotype analysis was performed using HAPSTAT software [18]. Cox proportional hazards regression was used to estimate the effect of SNPs on survival. SNPs that were significantly associated with survival in the univariate model were then reanalyzed with adjustment for patient age, tumor location, disease stage and treatment. For each hazard ratio (HR), a 95% confidence interval (95% CI) was calculated. P-values less than 0.05 were considered statistically significant. Interactions between SNPs and treatment protocols (5FU+cisplatin) were examined using the addition of interaction terms in the Cox model. The false discovery rate (FDR) method [19] was applied to address multiple comparisons. FDR was applied based on the number of independent SNPs within each gene [20] and the number of genes related to each hypothesis. The sample size and design of this study allows detection of HRs of 2.1 or more with 80% statistical power for a MAF≥30%.

### Gel Shift Assays

4 µM double-stranded probes were made by heating 200 pmol each of HPLC-purified forward and reverse oligo in 50 uL of Tris:EDTA to 90°C and cooling to room temperature. 4 pmol of each double-stranded probe was radioactively labeled with 10 µCi [γ-P32]ATP (Perkin Elmer, Waltham, MA) in a 10 uL reaction with 10 units T4-PNK (Promega, Madison, WI) and 1X T4-PNK buffer (Promega, Madison, WI). Labeled probes were diluted to 0.08 pmol/uL in Tris:EDTA and cleaned in an illustra ProbeQuant G-50 micro column (GE Healthcare Life Sciences, Buckinghamshire, UK ). 10 uL binding reactions included 1x Gel Shift binding buffer (Promega, Madison, WI) and ∼12 ug HeLaScribe Nuclear extract (Promega, Madison, WI), with 4 pmol unlabelled probe (or Tris:EDTA), pre-incubated at room temperature for 5 minutes before the addition of 0.08 pmol (∼20 000 cpm) labeled probe, followed by a 20 minute incubation at room temperature. Samples were separated on a Novex 6% DNA retardation gel (Life Technologies, Burlington, ON) run at 100 V in 0.5X TBE for 1 hour. Gels were transferred to Whatman paper and dried for 2 hours at 80°C. Dried gels were exposed to a Fugifilm Imaging Plate (Fugifilm, Mississauga, ON) for ∼18 hours and images captured on a Fugifilm FLA-7000 scanner (Fugifilm, Mississauga, ON).

## Results

### Characteristics of the Patients

During the study period, *202* gastroesophageal junction (GEJ) adenocarcinomas patients were assessed for eligibility. Excluded cases were *15 (27%)* patients who were already deceased at the time of assessment, *18 (33%)* patients who were already deceased at time of contact and *22 (40%)* patients who were unable to consent because of poor health. The total number of eligible cases for the study was *147* patients. Of these, *4 (3%)* could not be contacted, *31 (21%)* did not reply to repeated study invitations, and *8 (5%)* refused to participate, leaving *104 (71%)* patients who were both eligible and willing to participate. Biological samples appropriate for DNA extraction were received for *94* cases. *4* cases were excluded based on ethnicity, leaving *90* samples for genotyping. Genotyping results were obtained for *88 (98%)* samples; genotype data from 85 samples met quality control criteria and were analyzed.

The median age of diagnosis was *63* years. Men accounted for *91%* of cases. With regard to BMI classifications, *27%* of cases were normal, *43%* were overweight and *30%* were obese. About half of the patients *(48%)* were diagnosed with a tumor in the esophagus (Siewert I); the others *(52%)* had a tumor in the GEJ. The majority of patients received chemotherapy or radiation *(65%)* as their primary treatment; *45%* underwent surgery before recruitment. In combination, *12.9%* of cases received chemotherapy+surgery+radiation; *29.4%* received chemotherapy+radiation; *16.5%* received chemotherapy+radiation; *7.1%* received radiation and surgery; *7.1%* received only chemotherapy; *15.3%* received only radiation; *8.2*% received only surgery and *3.5%* received no treatment. Stage was assessed for all patients: *59%* had local/regional disease and *41%* had metastatic disease. **Table 1** shows the demographic and clinical characteristics of the cohort and their association with survival of patients. Chemotherapy is the only treatment that shows a statistically significant association with survival.

**Table 1:** Demographic and clinical features of the cohort by survival status.

Feature	Category	Alive	Dead	HR 95% CI
Sex	Women	4(50.0%)	4(50.0%)	0.98 (0.349, 2.746)
	Men	38(49.4%)	39(50.6%)	
Age Group	65>	27(57.4%)	20(42.6%)	1.51 (0.83, 2.75)
	65 and more	15(39.5%)	23(60.5%)	
BMI	Normal(18.5–24.9)	12(54.5%)	10(45.5%)	0.924 (0.629, 1.357)
	overweight(25–29.9)	12(34.3%)	23(65.7%)	
	overweight(25–29.9)	12(34.3%)	23(65.7%)	
Location of tumor	Siewert I	21(51.2%)	20(48.8%)	1.24 (0.68, 2.27)
	Siewert II	21(47.7%)	23(52.3%)	
Stage	IA	2(66.7%)	1(33.3%)	1.16 (0.98, 1.38)
	IIA	1(50.0%)	1(50.0%)	
	IIB	11(68.8%)	5(31.3%)	
	IIIA	13(61.9%)	8(38.1.0%)	
	IIIB	0(0.0%)	5(100.0%)	
	IIIC	2(66.7%)	1(33.3%)	
	IV	13(37.1%)	22(62.9%)	
Metastatic	No	29(58.0%)	21(42.0%)	1.60 (0.89, 2.92)
	Yes	13(37.1%)	22(62.9%)	
Chemotherapy	No	8(26.7%)	22(73.3%)	*0.37 (0.20, 0.68)*
	Yes	34(61.8%)	21(38.2%)	
Radiation	No	18(60.0%)	12(40.0%)	1.56 (0.80, 3.07)
	Yes	24(43.6%)	31(56.4%)	
Surgery	No	19(40.4%)	28(59.6%)	0.54(0.29, 1.01)
	Yes	23(60.5%)	15(39.5%)	

### Associations of SNPs with Survival

**Figure 1:**
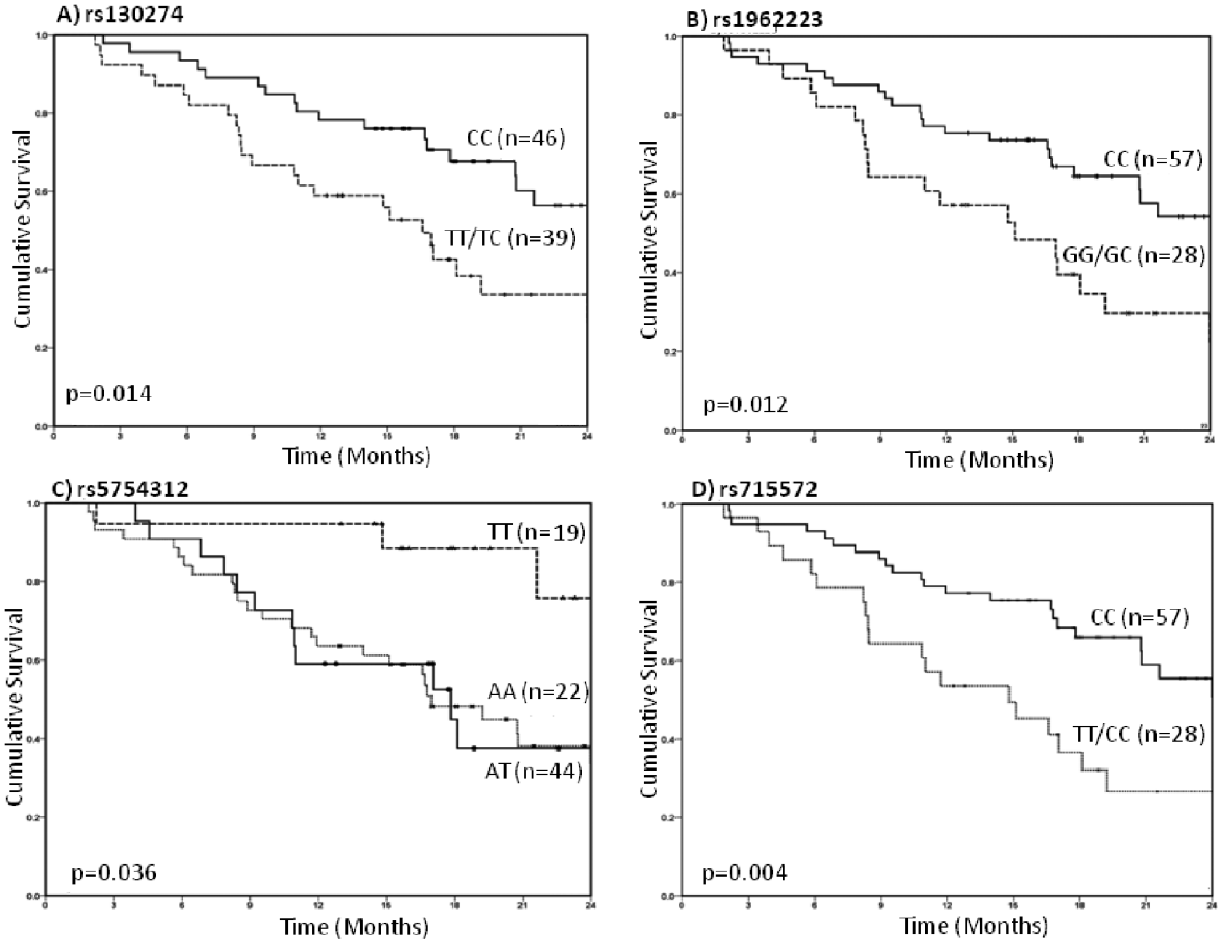
Survival of the study cohort by *TIMP3* variations. Kaplan-Meier survival curves and log-rank test p-values are shown. **A**) rs130274, **B**) rs1962223, **C**) rs5754312, **D**) rs715572.

At the end of follow-up, *51%* events (deaths) had occurred. Cancer was the cause of death for all patients. Kaplan-Meier survival curves and log-rank test p-values for SNPs associated with survival (rs130274, rs1962223, rs5754312 and rs715572) are shown in **Figure 1**.

**Figure 2:**
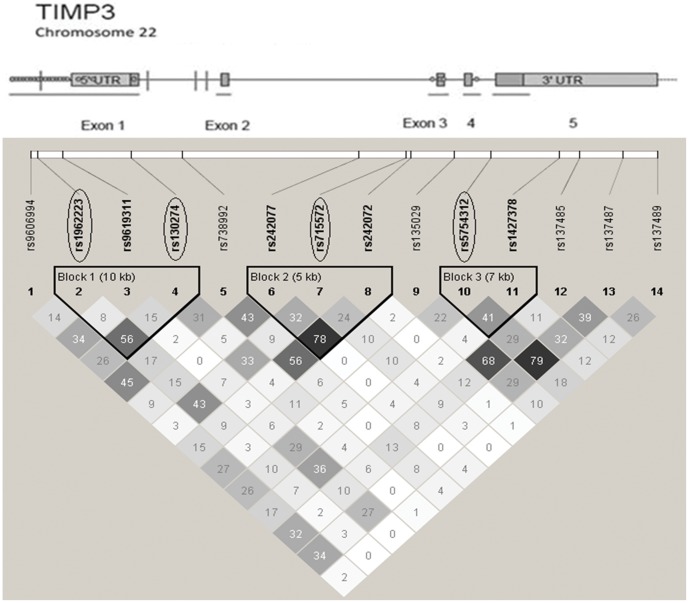
Structure of the *TIMP3* gene. Coding regions of exons are shown as dark bars; 5′ and 3′ untranslated regions are shown as lighter bars; introns and flanking sequence are illustrated by a line. The diagram is not to scale. Thick lines below the gene structure indicate the regions sequenced. SNPs associated with survival are indicated by vertical lines. Variants detected by sequencing are shown as circles. An LD plot based on study data is below the gene. Numbers in the plot are r^2^ values; boxes with darker shading illustrate higher LD; lighter shading represents weaker LD. Circled SNPs show significant association with survival.

Estimates of association from a Cox model of survival with all MMP and TIMP genetic variations are shown in **Table S3**. Univariate analysis showed that *TIMP3* SNPs were significantly associated with the survival of GEJ cancer patients. **Table 2** shows the survival model for *TIMP3* SNPs before and after adjusting for the patients’ age, tumor location, disease stage and treatment. The *14 TIMP3* SNPs tested and the linkage disequilibrium (LD) structure in patient data is shown in **Figure 2**. Of these, four (rs130274, rs1962223, rs715572 and rs5754312) were associated with survival both before and after adjustment for patient variables; p-values were more significant with adjustment (*0.0012, 0.0012, 0.0023* and *0.018,* respectively). Using the method of Nyholt [20], which is based on inter-SNP linkage disequilibrium (LD), the *14* SNPs in TIMP3 are equivalent to *11.8* independent SNPs. Three out of four survival-associated SNPs passed multiple testing correction using the FDR method [19] for *11.8* independent SNPs (*p = 0.013, 0.0067, 0.0073* for rs130274, rs1962223 and rs715572, respectively); the fourth SNP (rs5754312), showed borderline significance after correction *(p = 0.053).* The SNP with the lowest p-value (taken to represent the gene), remained significant after correction for the number of TIMP genes tested (4) *(p = 0.028)*, as well as for all 7 genes studied *(p = 0.049).*

**Table 2:** Hazard ratio (HR) and 95% Confidence intervals (CI) estimated for the association between *TIMP3* gene variations and survival of the study cohort.

			Unadjusted		Adjusted	
SNP ID	Alleles	Frequency (%)	HR (95% CI)	p	HR (95% CI)	p
**rs130274**	*CC*	46(54.1%)	1		1	
	*TC*	34(40.0%)	2.00(1.07, 3.75)		3.31 (1.60, 6.85)	
	*TT*	5(5.9%)	3.68(1.04, 12.97)		2.50 (0.67, 9.40)	
	*CC vs TT/TC*		2.11(1.15, 3.88)	*0.016*	3.15 (1.58, 6.30)	*0.001*
**rs135029**	*CC*	40(47.6%)	1		1	
	*TC*	36(42.9%)	1.16(0.60, 2.24)		1.12(0.56, 2.25)	
	*TT*	8(9.5%)	2.00(0.79, 5.08)		2.59(0.93, 7.02)	
	*CC vs TT/TC*		1.29(0.69, 2.40)	0.419	1.33 (0.70, 2.54)	0.38
**rs137485**	*AA*	42(50.0%)	1		1	
	*AT*	36(42.9%)	1.21(0.64, 2.28)		1.05 (0.53, 2.10)	
	*TT*	6(7.1%)	2.03(0.69, 6.00)		2.45 (0.78, 7.66)	
	*AA vs TT/AT*		1.30(0.71, 2.38)	0.403	1.21 (0.64, 2.30)	0.56
**rs137487**	*GG*	24(28.2%)	1		1	
	*AG*	44(51.8%)	2.09(0.78, 5.62)		2.30 (0.73, 7.21)	
	*AA*	17(20.0%)	2.31(1.00, 5.30)		2.16 (0.85, 5.48)	
	*GG vs AA/AG*		2.25(1.00, 5.05)	*0.05*	2.17 (0.86, 5.48)	0.09
**rs137489**	*AA*	48(56.5%)	1		1	
	*AG*	33(38.8%)	1.25(0.68, 2.32)		1.22 (0.63, 2.40)	
	*GG*	4(4.7%)	–		–	
	*AA vs GG/AG*		1.02(0.55, 1.89)	0.945	1.04 (0.54, 2.01)	0.9
**rs1427378**	*AA*	44(51.8%)	1		1	
	*AG*	35(41.2%)	0.22(0.03, 1.60)		0.96 (0.50, 1.86)	
	*GG*	6(7.1%)	0.96(0.52, 1.76)		0.18 (0.02, 1.46)	
	*AA vs GG/AG*		0.82(0.45, 1.49)	0.513	0.86 (0.44, 1.67)	0.65
**rs1962223**	*CC*	57(67.1%)	1		1	
	*CG*	26(30.1%)	2.75(1.40, 5.37)		2.97 (1.54, 5.75)	
	*GG*	2(2.4%)	–		–	
	*CC vs GG/CG*		2.16(1.17, 3.97)	*0.014*	2.97 (1.54, 5.75)	*0.001*
**rs242072**	*TT*	24(28.2%)	1		1	
	*TC*	40(47.1%)	0.93(0.46, 1.88)		1.06 (0.43, 2.56)	
	*CC*	21(24.7%)	0.80(0.35, 1.83)		1.17 (0.52, 2.68)	
	*TT vs CC/TC*		0.89(0.46, 1.70)	0.715	1.12 (0.53, 2.37)	0.76
**rs242077**	*CC*	29(34.5%)	1		1	
	*TC*	40(47.6%)	1.19(0.60, 2.37)		1.11 (0.49, 2.52)	
	*TT*	15(17.9%)	1.25(0.54, 2.90)		0.948 (0.40, 2.30)	
	*CC vs TT/TC*		1.21(0.64, 2.30)	0.555	1.84 (0.91, 3.71)	0.9
**rs5754312**	*AA*	22(25.9%)	1		1	
	*TA*	44(51.8%)	1.03(0.52, 2.05)		0.90 (0.44, 1.80)	
	*TT*	19(22.4%)	0.29(0.09, 0.89)		*0.23 (0.66, 0.82*)	
	*TT vs AA/TA*		0.28(0.1, 0.79)	*0.016*	0.25 (0.08, 0.79)	*0.018*
**rs715572**	*CC*	57(67.1%)	1		1	
	*TC*	24(28.2%)	2.53(1.36, 4.71)		2.67 (1.41, 5.08)	
	*TT*	4(4.7%)	1.49(0.35, 6.35)		2.54 (0.55, 11.7)	
	*CC vs TT/TC*		2.37(1.30, 4.32)	*0.005*	2.66 (1.418, 5.00)	*0.002*
**rs738992**	*CC*	20(23.5%)	1		1	
	*TC*	50(58.8%)	1.11(0.52, 2.37)		1.13 (0.52, 2.5)	
	*TT*	15(17.6%)	1.22(0.48, 3.09)		0.8 (0.28, 2.2)	
	*CC vs TT/TC*		1.13(0.543, 2.37)	0.737	1.05 (0.48, 2.28)	0.91
**rs9606994**	*GG*	26(30.6%)	1		1	
	*AG*	45(52.9%)	1.59(0.77, 3.28)		0.70 (0.24, 1.96)	
	*AA*	14(16.5%)	0.95(0.34, 2.62)		1.62 (0.74, 3.52)	
	*GG vs AA/AG*		1.41(0.70, 2.87)	0.338	1.27 (0.60, 2.70)	0.53
**rs9619311**	*AA*	43(50.6%)	1		1	
	*AG*	31(36.5%)	0.71(0.38, 1.34)		0.76 (0.39, 1.48)	
	*GG*	11(12.9%)	0.23(0.06, 0.99)		0.256 (0.06, 1.14)	
	*AA vs GG/AG*		0.58(0.313, 1.07)	0.079	0.29 (0.07, 1.25)	0.09
**rs9862**	*CC*	25(29.4%)	1		1	
	*TC*	42(49.4%)	2.85(1.23, 6.60)		2.75(1.07, 7.12)	
	*TT*	18(21.2%)	3.035(1.17, 7.87)		3.50(1.24, 9.90)	
	*CC vs TT/TC*		0.35(0.15, 0.77)	*0.01*	0.34(0.14, 0.84)	*0.02*

* Adjusted for patient age, disease stage, surgery, chemotherapy, radiation therapy, location of tumor.

rs1962223, which is near the promoter region of *TIMP3*, was associated with a 3-fold increased risk of death for patients who carried the CG genotype after adjustment for patient age, tumor location, disease stage and treatment. rs130274 is in moderate LD *(r^2^ = 0.56)* with rs1962223. rs130274 shows a more than *3*-fold increased risk of death. rs715572 was associated with about a *3*-fold increased HR; rs5754312 was associated with a 4-fold reduction in the HR. A haplotype including rs5754312 and rs715572 showed significant association with survival *(p = 0.002).* Our sample size did not permit additional tests for interactions between SNPs.

### Analysis for Interactions between *TIMP3* Genotypes and Chemotherapy

Interaction analyses revealed significant interactions between each of the four *TIMP3* survival-associated SNPs and chemotherapy. In stratified analyses, genotypes at rs715572 and rs5754312 (but not the other two SNPs) were significantly associated with outcome for patients who received chemotherapy, but not associated with outcome in patients who did not receive chemotherapy *HR = 2.7 (95% CI; 1.15, 6.4); HR = 0.13 (95% CI; 0.02, 0.95)* respectively.

### Re-sequencing *TIMP3* in the Study Samples

To identify and determine genotypes for additional *TIMP3* SNPs potentially also associated with GEJ cancer outcome, we performed Sanger sequencing of the coding exons, 5′ UTR and promoter region of *TIMP3*. Sequencing revealed 23 SNPs. A structural model of the gene and the locations of the SNPs detected is included in **Figure 2**. A summary of variants detected in *TIMP3* sequenced regions is in **Table S4.** SNPs with MAF >0.05 (10 out of 23 SNPs) were considered for analysis. Four of those 10 (rs9606994, rs1962223, rs9619311 and rs137485) had already been genotyped. Genotypes determined from the sequence data for these *4* SNPs were consistent with the original Sequenom genotypes. Of the other SNPs in *TIMP3* (rs62232902, rs8137129, rs5749511, rs2234921, rs9862 and rs11547635), only rs9862 showed association with survival (a 3-fold reduction in the HR), however, this SNP is in relatively high LD *(r^2^ = 0.77)* with one of the genotyped SNPs (rs5754312).

### Functional Analysis of *TIMP3* SNPs

None of the variants associated with survival in this study causes a deleterious protein coding change in *TIMP3*. Of the associated SNPs, only rs9862 in exon 3 is within the *TIMP3* coding region. Although this SNP does not result in an amino acid change, its location within the *TIMP3* transcript makes it a useful indicator SNP to assess allele-specific expression levels. Attempts to assess allelic imbalance by examining relative allele expression ratios in RNA extracted from cultured lymphoblasts were unsuccessful, however, due to extremely low *TIMP3* expression in lymphocytes (data not shown). Low TIMP3 expression in lymphocytes also prevents assessment of allelic imbalance using variants in the 3′UTR.

**Figure 3:**
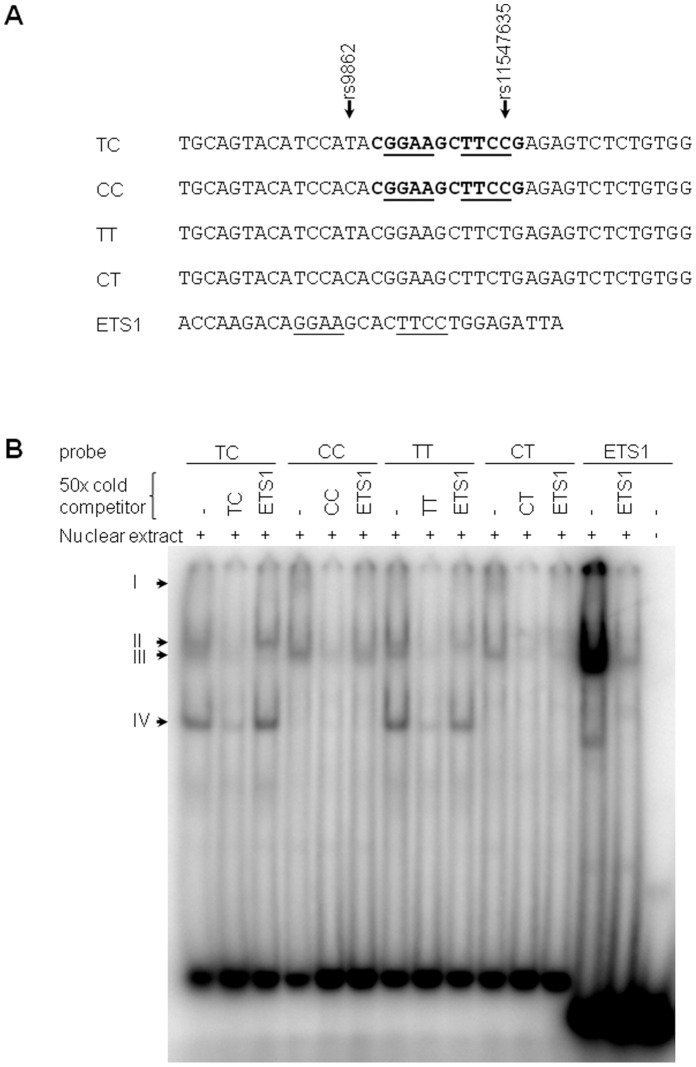
Gel shift assay for TIMP3 SNPs rs9862 and rs11547635. A) Probe names and sequences. rs9862 and rs11547635 are indicated with arrows. The 12 bp palindromic sequence is highlighted in bold and core ETS1 consensus sites are underlined. Probes are named according to alleles at sites rs9862 and rs11547635, respectively. Only one strand of the double-stranded probes is shown. B) Results. Probe and competitor names correspond to the sequences in A. Lane 15 is a control, with no nuclear extract. Potential protein complexes bound to the probes are indicated with letters on the left side of the image. Complex I appears to be specific to probes with the rs9862 C allele, whereas complexes II and IV are specific to probes with the rs9862 T allele. Complex III binds irrespective of rs9862 allele, and is competed off by unlabelled ETS1.

Although not associated with survival in this study, rs11547635 is also of functional interest because it is located within 12 bp of rs9862. rs11547635 disrupts an ETS1 consensus binding site and a 12 base pair palindromic sequence. To test whether these SNPs affect binding of protein factors to these sequences, probes differing by rs9862 and rs11547635 alleles (**Figure 3A**) were subjected to gel shift assays **(** **Figure 3B** **)**. rs9862 was found to influence protein binding in an allele specific manner. A protein complex (indicated as Complex I in **Figure 3B**) appears to be specific to probes with the rs9862 C allele, whereas Complexes II and IV are specific to probes with the rs9862 T allele. Complex III binds irrespective of rs9862 allele, and is competed off by unlabelled ETS1. Complexes II and IV were not competed off by an unlabelled ETS1 consensus competitor, suggesting that they are unlikely to be ETS1. No differences in binding to proteins in HeLa cell extracts were detected in probes differing by rs11547635 allele.

## Discussion

We have demonstrated the association of *TIMP3* polymorphisms with survival of GEJ adenocarcinomas. SNPs in three MMP genes of interest (*MMP2*, *MMP9* and *MMP7*) were not associated with survival; however, this negative finding should not be generalized to all members of the MMP gene family. TIMP gene products are natural inhibitors of the proteolytic activity of MMPs and adamalysins proteins [21]. The product of *TIMP3* is a 24-kDa protein that, unlike other TIMP protein family members, binds to the ECM [22]. The *TIMP3* gene acts as a tumor suppressor in some cancers by affecting tumor growth, angiogenesis, invasion and the development of metastases [9], [22], [23]. In addition to MMP inhibition, TIMP3 blocks the binding of vascular endothelial growth factor (VEGF) to the VEGF receptor-2, causing inhibition of angiogenesis [24] and has also been reported to induce apoptosis in cancer cells [25].

*TIMP3* methylation has been associated with cancer outcomes and response to treatment in a number of studies [26]–[30]. Cancer cell lines with methylated *TIMP3* have reduced *TIMP3* expression and are more sensitive to 5-FU than those with unmethylated *TIMP3* [31]. Loss of TIMP3 expression correlates with poor prognosis, supporting the involvement of TIMP3 in preventing tumor metastasis [30], [32]. SNPs in *TIMP3* have been associated with breast cancer prognosis [33], [34] however, this study is the first report of an association between *TIMP3* polymorphisms and survival of gastric or esophageal cancer patients.

Initial functional analyses suggest rs9862 may have a functional effect on the *TIMP3* gene. Initial tests for allelic imbalance in lymphoblast cell lines using rs9862 in exon 3 were inconclusive due to *TIMP3* expression levels too low for quantitation of allele-specific transcripts. This SNP and others in the 3′ UTR will be useful in the future, however, to assess allele-specific expression in tissue types directly relevant for GEJ adenocarcinoma, such as in microdissected patient tumor samples. Gel shift assays of rs9862 suggest that this SNP influences binding of an unidentified protein in an allele-specific manner, supporting the hypothesis that it may have a functional role. Identification of the differentially bound protein and investigation into its expression and role in GEJ adenocarcinoma may highlight the importance of *TIMP3* and this SNP in this cancer.

It is possible that rs9862 or one of the other variants associated with survival exerts a functional effect by influencing gene expression, splicing, epigenetic modification, or RNA stability of *TIMP3*. Because of the complex linkage disequilibrium structure in this region, however, it is also possible that an as yet undiscovered SNP (or SNPs) is responsible for the association. Such a SNP could be located outside the regions sequenced in our study. The fact that 2 of the associated variants, rs5754312 and rs9862, although in LD with each other, are only in weak LD with the other survival-associated variants, is consistent with the hypothesis that it is an undiscovered variant or variants, in LD with the associated SNPs, that is functionally responsible for the observed association. In addition, only two of the four survival-associated SNPs in *TIMP3* are specifically associated with survival after chemotherapy. It is possible that the effect of *TIMP3* on survival is complex and could involve multiple SNP effects. A larger-scale and systematic functional characterization of *TIMP3* genetic variants, in relevant tissue types, will likely be necessary to reveal the molecular basis for the association of *TIMP3* SNPs with GEJ adenocarcinoma survival.

Our study has several strengths. A prospective design places the study in the context of current treatments for GEJ adenocarcinomas. This study includes patients from the entire province of BC. Treatment disparity is minimal among the participants because all BC residents are covered for healthcare through the BC Medical Services Plan (MSP). The GI Tumour Group at the BCCA provides care for all patients in the province and devises province-wide treatment guidelines and protocols. Our use of a candidate gene design addresses genetic pathways of known biological relevance, and is based on a prior hypothesis for each gene. This approach simplifies interpretation of findings based on the biological plausibility of each gene and minimizes loss of study power due to correction for multiple tests.

A limitation of this study is that our results do not apply to patients with very short survival (i.e. less than 2 months) or additional substantial health problems because such patients may have been too sick to consent for our study. Because of this limitation, our results do not apply to patients with very short survival. Compared to other cancers, adenocarcinoma of the GEJ is a rare disease; though a province-wide study, the number of cases accrued in this study did not allow us to detect HRs <2.1 or stratify patients based on more specific treatment groups. A consortium of research groups would be required to obtain enough samples to detect smaller predictive and prognostic effect sizes (i.e., smaller HRs) for this uncommon cancer. These results should be replicated in other studies.

Adenocarcinomas of the GEJ are deadly cancers that are often diagnosed at a stage when treatment options are limited and have limited effectiveness, therefore identifying genetic variants that predict survival and chemotherapy response for this cancer is particularly important. Although our results do not establish the biological mechanism by which TIMP3 affects survival, TIMP3 is involved in a variety of steps affecting cancer progression, including the induction of apoptosis [35] and anti-angiogenesis [36] possibly by directly binding to VEGF receptor 2 or inhibiting ADAM-17 activity [37]. Regardless of the mechanism, our results suggest that *TIMP3* genetic variants should be considered as promising prognostic and predictive factors for GEJ adenocarcinoma, and warrant further study.

## Supporting Information

Table S1 **SNPs in TIMP and MMP genes used for survival analyses.**
(PDF)

Table S2 **Primers for sequencing** ***TIMP3*** **: forward and reverse primer sequences are shown, as well as PCR product size and PCR conditions.**
(PDF)

Table S3 **Hazard ratios (HR) and 95% Confidence intervals (CI) estimates for the association between** ***TIMP*** **and** ***MMP*** **gene variations and survival (unadjusted).**
(PDF)

Table S4 **Summary of variants detected in** ***TIMP3*** **regions sequenced.**
(PDF)
