# Supplementary material for: Genetic Polymorphisms at TIMP3 Are Associated with Survival of Adenocarcinoma of the Gastroesophageal Junction
Source: PLoS One. 2013 Mar 19;8(3):e59157. doi: 10.1371/journal.pone.0059157 (PMC3602604; doi:10.1371/journal.pone.0059157)
Supplement: Table S1 — SNPs in TIMP and MMP genes used for survival analyses. (PDF) [file pone.0059157.s001.pdf]

Supplementary table 1: SNPs in TIMP and MMP genes used for survival analyses.

Gene Name	Reference ID	Chromosome	Genomic position	Alleles	Location in Gene	HapMap MAF	HWE p*	GENOTYPE %*	MAF*	Alleles*
MMP2	rs11541998	16	54094264	C/G	SYNONYMOUS CODING	0.102	1	100	0.1	C/G
MMP2	rs11639960	16	54090771	A/G	INTRONIC	0.35	0.905	98.8	0.286	T/C
MMP2	rs17301608	16	54076111	C/T	INTRONIC	0.385	0.563	98.8	0.304	C/T
MMP2	rs1992116	16	54085392	G/A	INTRONIC	0.469	0.437	100	0.347	C/T
MMP2	rs243842	16	54084923	T/C	INTRONIC	0.36	0.324	100	0.418	T/C
MMP2	rs243847	16	54081499	T/C	INTRONIC	0.398	0.397	100	0.441	A/G
MMP2	rs243865	16	54069307	C/T	UPSTREAM	0.243	1	100	0.2	C/T
MMP2	rs7201	16	54097115	A/C	3PRIME UTR	0.451	0.945	97.6	0.355	T/G
MMP7	rs10502001	11	101903803	C/T	NON SYNONYMOUS CODING	0.205	0.074	100	0.253	G/A
MMP7	rs11225308	11	101904688	T/G	INTRONIC	0.21	0.056	98.8	0.25	A/C
MMP7	rs12184413	11	101894798	C/T	DOWNSTREAM	0.102	1	100	0.118	G/A
MMP7	rs12285347	11	101901817	T/C	INTRONIC	0.467	0.162	100	0.453	A/G
MMP7	rs1996352	11	101901457	C/T	INTRONIC	0.23	1	100	0.206	T/C
MMP7	rs495041	11	101895398	C/T	DOWNSTREAM	0.128	1	100	0.165	G/A
MMP7	rs880197	11	101910881	A/T	UPSTREAM	0.235	1	100	0.206	A/T
MMP9	rs17576	20	44073632	A/G	NON SYNONYMOUS CODING	0.363	0.153	100	0.353	A/G
MMP9	rs3918261	20	44076999	A/G	INTRONIC	0.164	1	100	0.165	T/C
TIMP1	rs4898	X	47329929	T/C	SYNONYMOUS CODING	0.485	0	100	0.494	C/T
TIMP1	rs6609533	X	47330230	A/G	INTRONIC	0.485	0	100	0.494	C/T
TIMP2	rs12452379	17	74427053	C/A	INTRONIC	0.496	1	100	0.429	G/T
TIMP2	rs12600817	17	74413060	G/A	INTRONIC	0.487	1	100	0.476	A/G
TIMP2	rs2277700	17	74378306	C/T	INTRONIC	0.254	1	100	0.188	T/C
TIMP2	rs2377004	17	74382054	C/T	INTRONIC	0.382	0.65	100	0.335	T/C
TIMP2	rs2889529	17	74409070	A/G	INTRONIC	0.429	0.749	100	0.459	A/G
TIMP2	rs4789932	17	74435870	G/A	UPSTREAM	0.442	0.447	98.8	0.393	C/T

Gene Name	Reference ID	Chromosome	Genomic position	Alleles	Location in Gene	HapMap MAF	HWE p*	GENOTYPE %*	MAF*	Alleles*
TIMP2	rs4789936	17	74409569	C/T	INTRONIC	0.496	1	100	0.471	A/G
TIMP2	rs6416835	17	74420830	A/G	INTRONIC	0.433	1	100	0.394	C/T
TIMP2	rs7211674	17	74410660	C/A	INTRONIC	0.438	0.779	100	0.447	A/C
TIMP2	rs7212662	17	74429726	T/G	INTRONIC	0.456	0.704	100	0.494	A/C
TIMP2	rs8064344	17	74388569	C/T	INTRONIC	0.25	1	100	0.188	T/C
TIMP2	rs8068674	17	74419040	C/T	INTRONIC	0.425	0.779	100	0.447	C/T
TIMP3	rs130274	22	31534334	C/T	INTRONIC	0.23	0.974	100	0.259	C/T
TIMP3	rs135029	22	31570290	A/G	INTRONIC	0.314	1	98.8	0.31	C/T
TIMP3	rs137485	22	31584283	T/A	INTRONIC	0.279	0.905	98.8	0.286	A/T
TIMP3	rs137487	22	31589104	A/G	DOWNSTREAM	0.473	0.907	100	0.459	G/A
TIMP3	rs137489	22	31592935	T/C	DOWNSTREAM	0.235	0.855	100	0.241	A/G
TIMP3	rs1427378	22	31582041	A/G	INTRONIC	0.254	1	100	0.276	A/G
TIMP3	rs1962223	22	31523905	G/C	UPSTREAM	0.183	0.139	100	0.165	C/G
TIMP3	rs2040435	22	31593431	C/T	DOWNSTREAM	0.288	1	100	0.165	C/A
TIMP3	rs242072	22	31565517	C/T	INTRONIC	0.478	0.712	100	0.482	T/C
TIMP3	rs242077	22	31559685	T/C	INTRONIC	0.392	0.984	98.8	0.417	C/T
TIMP3	rs5754312	22	31574421	A/T	INTRONIC	0.473	0.948	100	0.482	A/T
TIMP3	rs715572	22	31564931	G/A	INTRONIC	0.221	0.657	100	0.188	C/T
TIMP3	rs738992	22	31540005	C/T	INTRONIC	0.5	0.162	100	0.471	C/T
TIMP3	rs9606994	22	31523050	G/A	UPSTREAM	0.483	0.642	100	0.429	G/A
TIMP3	rs9619311	22	31526693	T/C	UPSTREAM	0.292	0.235	100	0.312	A/G
TIMP4	rs308952	3	12129428	A/G	INTRONIC	0.107	1	97.6	0.133	G/A
TIMP4	rs3755724	3	12175906	C/T	REGULATORY REGION	0.35	0.898	100	0.341	G/A

*Observed in the study cohort. When different alleles are reported between the dbSNP entry and study data, it is because the opposite strand was assayed.
