# Supplementary material for: Genetic Polymorphisms at TIMP3 Are Associated with Survival of Adenocarcinoma of the Gastroesophageal Junction
Source: PLoS One. 2013 Mar 19;8(3):e59157. doi: 10.1371/journal.pone.0059157 (PMC3602604; doi:10.1371/journal.pone.0059157)
Supplement: Table S2 — Primers for sequencing TIMP3 : forward and reverse primer sequences are shown, as well as PCR product size and PCR conditions. (PDF) [file pone.0059157.s002.pdf]

Supplementary Table 2: Primers used for sequencing TIMP3: forward and reverse directions plus product size and sequencing extension

Amplicon	Location	Primer Pair	Forward primer*	Reverse primer*	Product size	Tann (^{o}C)
1	upstream	1817F.1 / 1817R.1	TGTAAAACGACGGCCAGTACGGGATGTTGACTGAGAGC	CAGGAAACAGCTATGACGAATCAATGCCGAAAACAGAA	698	57.0
2	upstream	1818F.1 / 1818R.1	TGTAAAACGACGGCCAGTCAAGAGACAAACAAGCAGGTG	CAGGAAACAGCTATGACTCGTTTGAGTCTTTGTACAGTGC	698	57.0
3	upstream	1819F.1 / 1819R.1	TGTAAAACGACGGCCAGTGGAGGACAAACCTGTATTGCTT	CAGGAAACAGCTATGACTCTGGCCTGTCTACACACCT	448	57.0
4	upstream	1820F.1 / 1820R.1	TGTAAAACGACGGCCAGTTGAGGTCCTTTTTACGACTTCTC	CAGGAAACAGCTATGACAAGCTAGGTGGGGTGAACCT	592	57.0
5	upstream	1821F.1 / 1821R.1	TGTAAAACGACGGCCAGTCGGAGTCTCACTCCATCACC	CAGGAAACAGCTATGACCTTCAAGCAGCAGTTTTCTCA	754	57.0
6	upstream	1822F.1 / 1822R.1	TGTAAAACGACGGCCAGTGGGAGTGAGAAGAAATTAACCAAA	CAGGAAACAGCTATGACCCCTTTGAGCACTTTAGCCATA	750	57.0
7	upstream	1823F.1 / 1823R.1	TGTAAAACGACGGCCAGTAGGCCACAGTTAGAGTGAAGG	CAGGAAACAGCTATGACGGGTGAGCTGATCCAAAACT	718	57.0
8	upstream	1824F.1 / 1824R.1	TGTAAAACGACGGCCAGTCAAAGCAGAATCAAGATGTCAA	CAGGAAACAGCTATGACTTGACTGTGCTTGGTGGAAC	738	54.2**
9	upstream	1825F.1 / 1825R.1	TGTAAAACGACGGCCAGTACAGCAGATGGCTTCCCATA	CAGGAAACAGCTATGACCTGCTCGCCTCTCCAAAGT	839	57.0
10	Exon_1	1826F.1 / 1826R.1	TGTAAAACGACGGCCAGTCCTCCAGCTCCTGCTCCT	CAGGAAACAGCTATGACTTTCCCCACCCTTCTCTTCT	699	57.0
11	Exon_2	1827F.1 / 1827R.1	TGTAAAACGACGGCCAGTGGTTTTGGATTATGGTAAAGTCACA	CAGGAAACAGCTATGACTGGGAAGATTAAAGCCCAGA	744	57.0
12	Exon_3	1828F.1 / 1828R.1	TGTAAAACGACGGCCAGTATTCCGCTTAGCAAAACGAC	CAGGAAACAGCTATGACTTCAAAGACGGGCCTAACAG	744	57.0
13	Exon_4	1829F.1 / 1829R.1	TGTAAAACGACGGCCAGTCATTATAAAGGGAGTGAAGAGTTGG	CAGGAAACAGCTATGACTGGGAGAACCTGCCAGTG	745	57.0
14.2	Exon_5	1834F.1 / 1834R.1	TGTAAAACGACGGCCAGTTCCAACCTAGGGCAGCTAGA	CAGGAAACAGCTATGACTTAGTGTCCAAGGGAAGCTCA	657	57.0
15	Exon_5	1835F.1 / 1835R.1	TGTAAAACGACGGCCAGTCATGCTCTCCAATTTCGGTTA	CAGGAAACAGCTATGACCAAGAGGGGTGGGAATTACA	498	57.0

*M13 forward and reverse sequencing extensions are shown in smaller type; specific primer sequences are in larger type.

PCR Conditions: 30 cycles (30 sec @ 94oC, 30 sec @ 57oC and 1min @ 68oC) **amplicon 8 had Tann of 54.2oC
