# Supplementary material for: Genetic Polymorphisms at TIMP3 Are Associated with Survival of Adenocarcinoma of the Gastroesophageal Junction
Source: PLoS One. 2013 Mar 19;8(3):e59157. doi: 10.1371/journal.pone.0059157 (PMC3602604; doi:10.1371/journal.pone.0059157)
Supplement: Table S3 — Hazard ratios (HR) and 95% Confidence intervals (CI) estimates for the association between TIMP and MMP gene variations and survival (unadjusted). (PDF) [file pone.0059157.s003.pdf]

Supplementary Table 3: Hazard ratios (HR) and 95% Confidence intervals (CI) estimates for the association between TIMP and MMP gene variations and survival (unadjusted)

Gene	SNP ID	Alleles	NO (Freq)		p
MMP2	rs11541998	CC	69(81.2%)	1	
		GG	15(17.6%)	3183.68(0.00, E)	
		CG	1(1.2%)	2502.46(0.00, E)	
		CC vs GG/CG		0.747 (0.315, 1.773)	0.508
	rs11639960	TT	42(50.0%)	1	
		TC	36(42.9%)	0.74(0.39, 1.39)	
		CC	6(7.1%)	0.45(0.11, 1.92)	
		TT vs TC/CC		1.931 (0.466, 8.005)	0.365
	rs17301608	CC	39(46.4%)	1	
		TC	39(46.4%)	0.92(0.50, 1.70)	
		TT	6(7.1%)	0.64(0.15, 2.74)	
		CC vs TC/TT		0.884 (0.485, 1.609)	0.686
	rs1992116	CC	34(40.0%)	1	
		TC	43(50.6%)	0.85(0.45, 1.60)	
		TT	8(9.4%)	0.76(0.26, 2.24)	
		CC vs TC/TT		0.835 (0.455, 1.533)	0.561
	rs243842	CC	26(30.6%)	1	
		TC	47(55.3%)	0.52(0.26, 1.02)	
		TT	12(14.1%)	1.33(0.59, 3.02)	
		CC vs TC/TT		0.502 (0.24, 1.049)	0.067
	rs243847	AA	24(28.2%)	1	
		AG	47(55.3%)	0.51(0.26, 1.01)	
		GG	14(16.5%)	1.18(0.53, 2.64)	
		AA vsAG/GG		0.552 (0.272, 1.123)	0.101
	rs243865	CC	54(63.5%)	1	
		TC	28(32.9%)	0.80(0.41, 1.57)	
		TT	3(3.5%)	0.92(0.12, 6.86)	
		CC vs TC/TT		0.811 (0.421, 1.56)	0.53
	rs7201	AA	35(42.2%)	1	
		AC	37(44.6%)	0.84(0.44, 1.61)	
		CC	11(13.3%)	0.75(0.28, 2.01)	
		AA vs AC/CC		1.226 (0.481, 3.124)	0.669
MMP7	rs10502001	GG	51(60.0%)	1	
		AG	25(29.4%)	0.99(0.50, 1.97)	
		AA	9(10.6%)	0.92(0.32, 2.64)	
		GG vs AG/AA		0.97 (0.52, 1.81)	0.93
	rs11225308	CC	51(60.7%)	1	
		AC	24(28.6%)	1.21(0.62, 2.36)	

Gene	SNP ID	Alleles	NO (Freq)		p
MMP7	rs11225308	AA	9(10.7%)	0.94(0.33, 2.71)	
		CC vs AC/AA		1.126 (0.402, 3.157)	0.821
	rs12184413	GG	66(77.6%)	1	
		AG	18(21.2%)	0.58(0.25, 1.38)	
		AA	1(1.2%)	8.01(1.01, 63.45)	
		GG vs AG/AA		0.669 (0.298, 1.505)	0.331
	rs12285347	AA	29(34.1%)	1	
		AG	35(41.2%)	1.37(0.69, 2.74)	
		GG	21(24.7%)	0.68(0.28, 1.66)	
		AA vs AG/GG		1.765 (0.815, 3.822)	0.15
	rs1996352	TT	53(62.4%)	1	
		TC	29(34.1%)	0.97(0.51, 1.83)	
		CC	3(3.5%)	0.55(0.07, 4.10)	
		TT vs TC/CC		1.788 (0.244, 13.087)	0.567
	rs495041	GG	59(69.4%)	1	
		AG	24(28.2%)	0.84(0.42, 1.68)	
		AA	2(2.4%)	1.69(0.23, 12.58)	
		GG vs AG/AA		0.879 (0.451, 1.714)	0.705
	rs880197	AA	53(62.4%)	1	
		TA	29(34.1%)	0.97(0.51, 1.83)	
		TT	3(3.5%)	0.55(0.07, 4.10)	
		AA vs TA/TT		0.926 (0.497, 1.724)	0.808
MMP9	rs17576	AA	32(37.6%)	1	
		AG	46(54.1%)	1.21(0.63, 2.33)	
		GG	7(8.2%)	1.23(0.41, 3.76)	
		AA vs AG/GG		0.911 (0.325, 2.554)	0.85
	rs2274755	CC	59(69.4%)	1	
		AC	24(28.2%)	0.61(0.30, 1.25)	
		AA	2(2.4%)	0.00(0.00, E)	
		CC vs AC/AA		0.556 (0.273, 1.13)	0.1
	rs3918261	TT	59(69.4%)	1	
		TC	24(28.2%)	0.61(0.30, 1.25)	
		CC	2(2.4%)	0.00(0.00, E)	
		TT vs TC/CC		21.256 (0.012, 37190.142)	0.42
TIMP1	rs4898	CC	40(47.1%)	1	
		TC	6(7.1%)	1.09(0.32, 3.66)	
		TT	39(45.9%)	1.05(0.57, 1.95)	
		CC vs TC/TT		1.055 (0.579, 1.922)	0.86

Gene	SNP ID	Alleles	NO (Freq)		p
TIMP1	rs6609533	CC	40(47.1%)	1	
		TC	6(7.1%)	1.09(0.32, 3.66)	
		TT	39(45.9%)	1.05(0.57, 1.95)	
		CC vs TC/TT		1.055 (0.579, 1.922)	0.86
TIMP2	rs12452379	GG	28(32.9%)	1	
		TT	41(48.2%)	1.39(0.70, 2.79)	
		TG	16(18.8%)	1.08(0.42, 2.74)	
		GG vs TT/TG		1.307 (0.67, 2.551)	0.432
	rs12600817	AA	23(27.1%)	1	
		AG	43(50.6%)	0.91(0.45, 1.82)	
		GG	19(22.4%)	0.92(0.39, 2.16)	
		AA vs AG/GG		1.022 (0.489, 2.136)	0.953
	rs2277700	TT	56(65.9%)	1	
		TC	26(30.6%)	1.02(0.53, 1.96)	
		CC	3(3.5%)	3.24(0.75, 13.94)	
		TT vs TC/CC		0.31 (0.073, 1.314)	0.112
	rs2377004	TT	36(42.4%)	1	
		TC	41(48.2%)	1.25(0.66, 2.36)	
		CC	8(9.4%)	1.56(0.52, 4.72)	
		TT vs TC/CC		0.725 (0.257, 2.043)	0.543
	rs2889529	AA	26(30.6%)	1	
		AG	40(47.1%)	1.31(0.64, 2.71)	
		GG	19(22.4%)	1.32(0.56, 3.12)	
		AA vs AG/GG		0.898 (0.441, 1.826)	0.766
	rs4789932	CC	33(39.3%)	1	
		TC	36(42.9%)	0.78(0.40, 1.50)	
		TT	15(17.9%)	0.67(0.27, 1.69)	
		CC vs TC/TT		0.746 (0.404, 1.377)	0.348
	rs4789936	AA	24(28.2%)	1	
		AG	42(49.4%)	0.83(0.42, 1.65)	
		GG	19(22.4%)	0.88(0.38, 2.03)	
		AA vs AG/GG		1.022 (0.489, 2.136)	0.953
	rs6416835	CC	31(36.5%)	1	
		TC	41(48.2%)	1.30(0.68, 2.50)	
		TT	13(15.3%)	0.82(0.30, 2.25)	
		CC vs TC/TT		1.178 (0.628, 2.206)	0.61
	rs7211674	AA	27(31.8%)	1	
		AC	40(47.1%)	1.61(0.77, 3.38)	
		CC	18(21.2%)	1.81(0.75, 4.35)	
		AA vs AC/CC		0.753 (0.37, 1.533)	0.434

Gene	SNP ID	Alleles	NO (Freq)		p
TIMP2	rs7212662	AA	23(27.1%)	1	
		AC	40(47.1%)	1.65(0.77, 3.55)	
		CC	22(25.9%)	1.13(0.46, 2.79)	
		AA vs AC/CC		1.234 (0.607, 2.509)	0.561
	rs8064344	TT	56(65.9%)	1	
		TC	26(30.6%)	1.02(0.53, 1.96)	
		CC	3(3.5%)	3.24(0.75, 13.94)	
		TT vs TC/CC		0.31 (0.073, 1.314)	0.112
	rs8068674	CC	27(31.8%)	1	
		TC	40(47.1%)	1.01(0.49, 2.05)	
		TT	18(21.2%)	1.20(0.52, 2.79)	
		CC vs TC/TT		1.062 (0.544, 2.072)	0.861
TIMP3	rs130274	CC	46(54.1%)	1	
		TC	34(40.0%)	2.00(1.07, 3.75)	
		TT	5(5.9%)	3.68(1.04, 12.97)	
		CC vs TT/TC		2.11(1.15, 3.88)	*0.016*
	rs135029	CC	40(47.6%)	1	
		TC	36(42.9%)	1.16(0.60, 2.24)	
		TT	8(9.5%)	2.00(0.79, 5.08)	
		CC vs TT/TC		1.29(0.69, 2.40)	0.419
	rs137485	AA	42(50.0%)	1	
		AT	36(42.9%)	1.21(0.64, 2.28)	
		TT	6(7.1%)	2.03(0.69, 6.00)	
		AA vs TT/AT		1.30(0.71, 2.38)	0.403
	rs137487	GG	24(28.2%)	1	
		AG	44(51.8%)	2.09(0.78, 5.62)	
		AA	17(20.0%)	2.31(1.00, 5.30)	
		GG vs AA/AG		2.25(1.00, 5.05)	*0.05*
	rs137489	AA	48(56.5%)	1	
		AG	33(38.8%)	1.25(0.68, 2.32)	
		GG	4(4.7%)	-	
		AA vs GG/AG		1.02(0.55, 1.89)	0.945
	rs1427378	AA	44(51.8%)	1	
		AG	35(41.2%)	0.22(0.03, 1.60)	
		GG	6(7.1%)	0.96(0.52, 1.76)	
		AA vs GG/AG		0.82(0.45, 1.49)	0.513
	rs1962223	CC	57(67.1%)	1	
		CG	26(30.1%)	2.75(1.40, 5.37)	
		GG	2(2.4%)	-	
		CC vs GG/CG		2.16(1.17, 3.97)	*0.014*

Gene	SNP ID	Alleles	NO (Freq)		p
TIMP3	rs242072	TT	24(28.2%)	1	
		TC	40(47.1%)	0.93(0.46, 1.88)	
		CC	21(24.7%)	0.80(0.35, 1.83)	
		TT vs CC/TC		0.89(0.46, 1.70)	0.715
	rs242077	CC	29(34.5%)	1	
		TC	40(47.6%)	1.19(0.60, 2.37)	
		TT	15(17.9%)	1.25(0.54, 2.90)	
		CC vs TT/TC		1.21(0.64, 2.30)	0.555
	rs5754312	AA	22(25.9%)	1	
		TA	44(51.8%)	1.03(0.52, 2.05)	
		TT	19(22.4%)	0.29(0.09, 0.89)	
		TT vs AA/TA		0.28(0.1, 0.79)	*0.016*
	rs715572	CC	57(67.1%)	1	
		TC	24(28.2%)	2.53(1.36, 4.71)	
		TT	4(4.7%)	1.49(0.35, 6.35)	
		CC vs TT/TC		2.37(1.30, 4.32)	*0.005*
	rs738992	CC	20(23.5%)	1	
		TC	50(58.8%)	1.11(0.52, 2.37)	
		TT	15(17.6%)	1.22(0.48, 3.09)	
		CC vs TT/TC		1.13(0.543, 2.37)	0.737
	rs9606994	GG	26(30.6%)	1	
		AG	45(52.9%)	1.59(0.77, 3.28)	
		AA	14(16.5%)	0.95(0.34, 2.62)	
		GG vs AA/AG		1.41(0.70, 2.87)	0.338
	rs9619311	AA	43(50.6%)	1	
		AG	31(36.5%)	0.71(0.38, 1.34)	
		GG	11(12.9%)	0.23(0.06, 0.99)	
		AA vs GG/AG		0.58(0.313, 1.07)	0.079
TIMP4	rs308952	GG	62(74.7%)	1	
		AG	20(24.1%)	1.26(0.65, 2.44)	
		AA	1(1.2%)	0.00(0.00,E.)	
		GG vs AG/AA		1.219 (0.631, 2.356)	0.55
	rs3755724	GG	36(42.4%)	1	
		AG	40(47.1%)	0.80(0.42, 1.53)	
		AA	9(10.6%)	1.52(0.61, 3.83)	
		GG vs AG/AA		0.911 (0.498, 1.666)	0.76
