# Supplementary material for: Genetic Polymorphisms at TIMP3 Are Associated with Survival of Adenocarcinoma of the Gastroesophageal Junction
Source: PLoS One. 2013 Mar 19;8(3):e59157. doi: 10.1371/journal.pone.0059157 (PMC3602604; doi:10.1371/journal.pone.0059157)
Supplement: Table S4 — Summary of variants detected in TIMP3 regions sequenced. (PDF) [file pone.0059157.s004.pdf]

Supplementary Table 4: Variants detected in *TIMP3* regions sequenced

SNP Name*	SNP ID**	Sequence Context	MAF***	Amino acid change
TIMP3_UPSTR(-4938)_G/A	rs9606994	AAAGGCACCC(G/A)CACCCCACCG	0.41	
TIMP3_UPSTR(-4083)_G/C	rs1962223	ACACAGAAGT(G/C)GACGCCTGGA	0.16	
TIMP3_UPSTR(-3918)_A/T	no rs #	TATGTTTGGA(A/T)TGCATTAGCT	0.01	
TIMP3_UPSTR(-3862)_G/A	rs118060913	AGATAAACCT(G/A)TCAGGAGGAC	0.01	
TIMP3_UPSTR(-3826)_C/A	rs62232902	CTGATTGGCT(C/A)AGAGGGTGAT	0.06	
TIMP3_UPSTR(-3323)_T/C	rs8137129	AGCCTGCATC(T/C)TCATTCTTTT	0.33	
TIMP3_UPSTR(-2645)_C/T	no rs #	AAGGTAATTT(C/T)GTTACTTTTC	0.01	
TIMP3_UPSTR(-1797)_DEL(CT)	no rs #	TGCATTTCTC(CT/-)CTGTTTCTAT	0.01	
TIMP3_UPSTR(-1604)_C/T	rs5749511	AAAGGGGTGA(C/T)GAGTTCCTGG	0.08	
TIMP3_UPSTR(-1295)_T/C	rs9619311	AGGGTGGAGC(T/C)CTGTCAGCCA	0.34	
TIMP3_UPSTR(-1204)_C/T	no rs #	GCTGTTCCCC(C/T)TGCCTGGTAC	0.01	
TIMP3_UPSTR(-1082)_C/G	no rs #	AGGCCCTCCC(C/G)AGTCATGTCC	0.01	
TIMP3_UPSTR(-914)_A/G	rs2234921	TTGCTCTGGG(A/G)GAGCACAGAA	0.34	
TIMP3_UPSTR(-898)_T/A	rs2234920	CAGAAAACAG(T/A)CTTCTATCAT	0.01	
TIMP3_UPSTR(-677)_G/T	no rs #	CTGTGGAAGC(G/T)GTCCTGCTGG	0.01	
TIMP3_UPSTR(-533)_G/A	no rs #	ACCGGTCCCG(G/A)GCGCGCCCCA	0.01	
TIMP3_UPSTR(-270)_G/T	no rs #	GGCGGCGGGC(G/T)CTCAGACGGC	0.01	
TIMP3_UPSTR(-197)_INS(CCGCCCGCCGAGTCC)	no rs #	CCGCCGAGTC(-/CCGCCCGCCGAGTCC)CTGCGCCAGC	0.01	
TIMP3_x1(7)_C/T	no rs #	GGCAATGACC(C/T)CTTGGCTCGG	0.01	**P to S**
TIMP3_IVS2(-154)_A/G	no rs #	TATCTTGGCT(A/G)TTGTGAATAT	0.01	
TIMP3_x3(249)_T/C	rs9862	AGTACATCCA(T/C)ACGGAAGCTT	0.43	
TIMP3_x3(261)_C/T	rs11547635	CGGAAGCTTC(C/T)GAGAGTCTCT	0.08	
TIMP3_IVS4(+158)_T/A	rs137485	ATAGTAAGGA(T/A)TGTTGCCCCA	0.27	

*relative to NM_000362.4

**Variants with no SNP ID were not seen in dbSNP build 135

***MAF in the samples sequenced
